# High Frequency Stimulation of the Pelvic Nerve Inhibits Urinary Voiding in Anesthetized Rats

**DOI:** 10.3389/fphys.2017.00623

**Published:** 2017-08-28

**Authors:** Jonathan J. Crook, Thelma A. Lovick

**Affiliations:** Physiology, Pharmacology and Neuroscience, University of Bristol Bristol, United Kingdom

**Keywords:** high frequency stimulation, pelvic nerve, micturition, rat, urinary continence

## Abstract

Urge Urinary Incontinence: “a sudden and uncontrollable desire to void which is impossible to defer” is extremely common and considered the most bothersome of lower urinary tract conditions. Current treatments rely on pharmacological, neuromodulatory, and neurotoxicological approaches to manage the disorder, by reducing the excitability of the bladder muscle. However, some patients remain refractory to treatment. An alternative approach would be to temporarily suppress activity of the micturition control circuitry at the time of need i.e., urgency. In this study we investigated, in a rat model, the utility of high frequency pelvic nerve stimulation to produce a rapid onset, reversible suppression of voiding. In urethane-anesthetized rats periodic voiding was induced by continuous infusion of saline into the bladder whilst recording bladder pressure and electrical activity from the external urethral sphincter (EUS). High frequency (1–3 kHz), sinusoidal pelvic nerve stimulation initiated at the onset of the sharp rise in bladder pressure signaling an imminent void aborted the detrusor contraction. Urine output was suppressed and tone in the EUS increased. Stimulating the right or left nerve was equally effective. The effect was rapid in onset, reversible, and reproducible and evoked only minimal “off target” side effects on blood pressure, heart rate, respiration, uterine pressure, or rectal pressure. Transient contraction of abdominal wall was observed in some animals. Stimulation applied during the filling phase evoked a small, transient rise in bladder pressure and increased tonic activity in the EUS, but no urine output. Suppression of micturition persisted after section of the contralateral pelvic nerve or after ligation of the nerve distal to the electrode cuff on the ipsilateral side. We conclude that high frequency pelvic nerve stimulation initiated at the onset of an imminent void provides a potential means to control urinary continence.

## Introduction

The urinary bladder operates in two modes: storage and voiding. During the storage phase whilst the bladder is filling, the detrusor muscle relaxes to accommodate the increase in fluid volume, whilst sustained contraction of the external urethral sphincter (EUS) induced by tonic activity in the pudendal nerves maintains urinary continence (Fowler et al., 2008; De Groat et al., 2015). The act of micturition is dependent on the functional integrity of central control circuitry, which permits voiding to occur only when it is safe and socially acceptable for the individual to do so. When the central micturition circuitry switches from storage to voiding mode, activity in parasympathetic pelvic nerve efferents initiates contraction of the detrusor muscle and pudendal nerve activity is inhibited so that the EUS relaxes to allow urine to exit through the urethra (Fowler et al., 2008; De Groat et al., 2015).

Disorders of bladder control may arise from malfunction at any stage of the control machinery. Urge urinary incontinence (UUI), “a sudden and uncontrollable desire to void which is impossible to defer” (Abrams et al., 2003) is extremely common (prevalence 13.3% for men; up to 30.0% for women, depending on age; Milsom et al., 2014) and is rated as the most bothersome of lower urinary tract symptoms (Agarwal et al., 2014). The condition may arise due to hyperexcitability of the detrusor muscle (overactive bladder). Alternatively, the control circuitry may become hyperexcitable and/or the facility to inhibit voiding in inappropriate social situations may fail. Current drug treatments for UUI are aimed at reducing the excitability of the bladder. These can be effective although undesirable side effects are not uncommon (Reynolds et al., 2015; Olivera et al., 2016). In patients refractory to pharmacological treatment intravesical injection of botulinum toxin may be offered, although this requires frequent re-injection for continued benefit. There is also a risk of urinary tract infection and high residual volume, which may require patients to perform self-catheterization (Gupta et al., 2015; Tubaro et al., 2015; Olivera et al., 2016; Truzzi et al., 2016).

Neuromodulation is another approach used to decrease the excitability of the overactive bladder. Percutaneous tibial nerve stimulation (PTNS) and sacral nerve stimulation (SNS) have both been adopted as clinical procedures for urge incontinence (Yamanishi et al., 2015). However, PTNS requires a frequent schedule of re-application whereas high levels of re-intervention are required with SNS and pain is a frequent side effect (Gupta et al., 2015; Tubaro et al., 2015; Olivera et al., 2016; Truzzi et al., 2016).

An alternative approach would be to suppress voids only when required i.e., at the onset of urge. Charge-balanced kilohertz frequency alternating current (KHFAC) has been shown to produce rapid onset, reversible conduction block in both myelinated and unmyelinated peripheral nerves (Joseph and Butera, 2009, 2011; Kilgore and Bhadra, 2014; Patel and Butera, 2015). We therefore considered whether KHFAC stimulation of the pelvic nerve might be employed to modulate urinary voiding. In a rat model we investigated whether KHFAC, initiated at the onset of an imminent involuntary void in rats, when humans would be expected to experience extreme urge sensation, could abort the void, and maintain urinary continence.

## Methods

The study conforms to the national guidelines for the care and use of animals and was carried out under the authority of UK Home Office Project License PPL30/3200 and approved by the Local Ethical Committee of the University of Bristol. Every effort was made to minimize the risk of animal's pain or suffering. At the end of the experiment the animals were killed by an overdose of anesthetic.

Female Wistar rats (*n* = 30, 196–254 g) were obtained from Charles River UK Ltd and housed at the University of Bristol Animal Services Unit. They were anesthetized with urethane (1.4 g kg^−1^ i.p.) and the right femoral artery and right femoral vein were cannulated to record, respectively, arterial blood pressure and heart rate and for infusion of fluids. The trachea was cannulated to maintain a patent airway and monitor respiratory airflow. Rectal temperature was maintained at 37°C by a homeothermic blanket system (Harvard Apparatus, Holliston, Massachusetts, USA). The depth of anesthesia was assessed throughout the experiment by monitoring the pedal reflex, blood pressure, and heart rate. If required, supplementary anesthesia and fluid replacement were administered via the cannula in the femoral vein.

**Figure 1:**
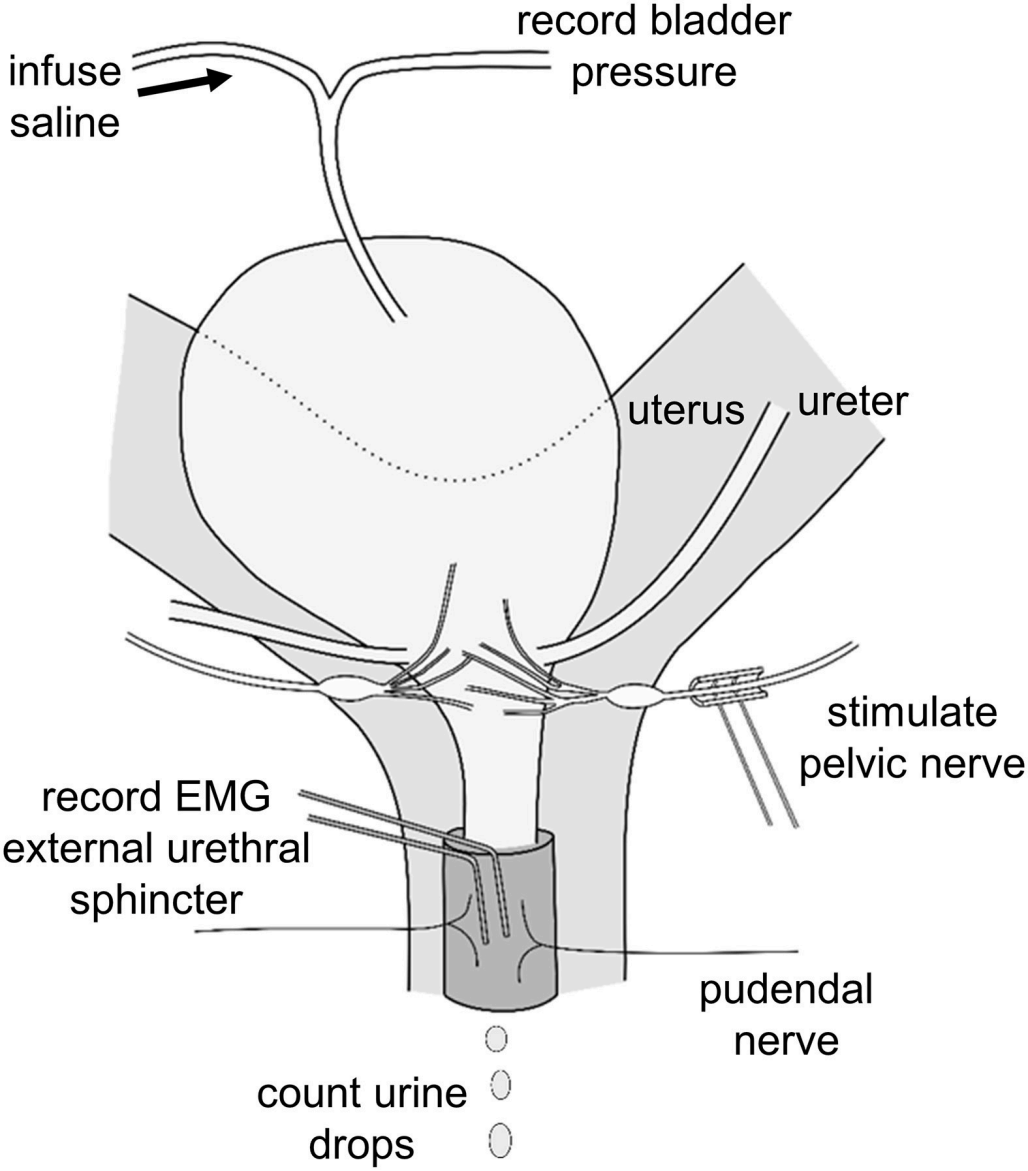
Schematic representation of location of stimulation and recording electrodes on pelvic nerve and external urethral sphincter and position of catheter in bladder used to record bladder pressure and infuse saline.

Animals were positioned supine, and a midline laparotomy was performed. The pelvic nerve was located and gently separated from the uterine wall. The distal portion of the preganglionic pelvic nerve bundle was fitted with a custom made bipolar cuff-electrode (platinum iridium wire with cobalt core in silicone epoxy; Figure 1). Stimulus trains of varying waveform, pulse duration, intensity and frequency were delivered using a stimulus isolation device (STMISOLA, Biopac Systems Inc., Santa Barbara, USA), driven by an alternating voltage waveform generator.

The bladder dome was cannulated by piercing it with a 25 G needle tip attached to a length of saline-filled polythene tubing. The tubing was attached to a T-piece, enabling recording of intravesical pressure during infusion of saline into the bladder. In 17 experiments two insulated platinum wire electrodes were inserted into the space between the pubic symphysis and the EUS muscle to record electromyogram (EMG) activity (Figure 1).

**Figure 2:**
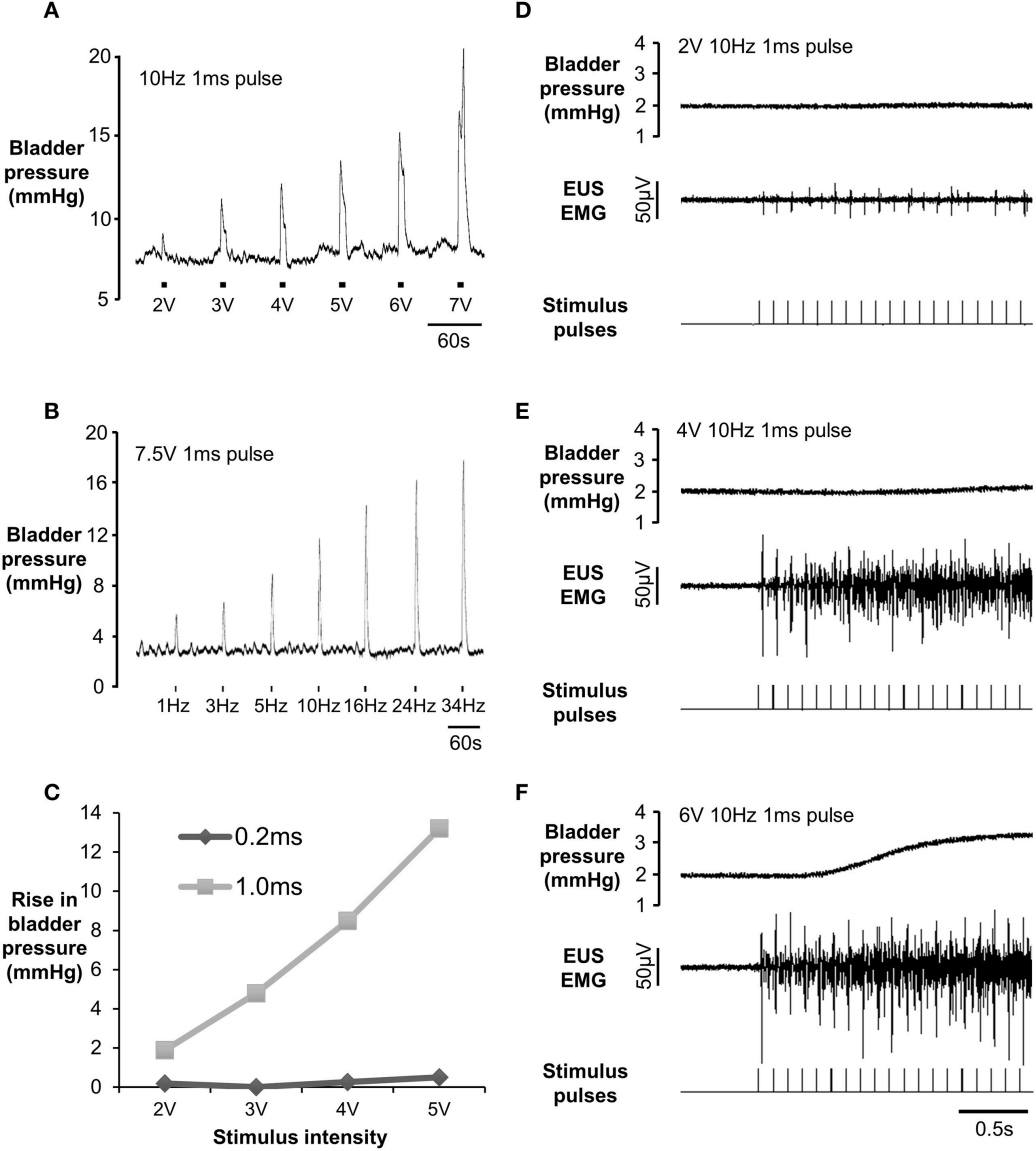
**(A)** Increase in bladder pressure evoked by low frequency unilateral electrical stimulation of the pelvic nerve (10 Hz, 1 ms pulses) at a range of intensities. **(B)** Frequency dependence of response evoked by 7.5 V 1 ms pulses, 10 Hz (150% of the voltage threshold at 10 Hz in this animal). **(C)** Effect of pulse duration on response to 10 Hz unilateral electrical stimulation of the pelvic nerve. **(D–F)** Bladder pressure (top trace) and EMG activity of external urethral sphincter (middle trace) in response to increasing intensity of stimulation. Stimulus artifacts on the EUS recording were digitally removed (see methods) to show twitch-like responses.

In five experiments two insulated stainless steel needle electrodes were inserted into the abdominal wall to record EMG of the abdominal muscles. EMG activity was amplified (5000x) using a Neurolog system (Digitimer Ltd, Welwyn Garden City, Hertfordshire, UK) and digitally bandpass filtered (0.1–500 Hz) offline, using Matlab R2014a. Stimulus artifacts present in the EMG signal resulting from low frequency pulse trains (e.g., Figures 2D–F), were excluded off-line by subtraction of the averaged stimulus-triggered waveform. Uterine and rectal pressures were monitored either by inserting a balloon catheter (2F Embolectomy catheter, Intra special catheters GmbH, Rehlingen-Siersburg, Germany) into the vagina and advancing it until the tip reached the uterus (*n* = 9), or into the rectum via the anus (*n* = 5). The timing of each drop of urine exiting the urethra was counted by visual observation and logged electronically. All data were captured and displayed using a PowerLab 8SP data acquisition system running Chart v5 software. Statistical tests were carried out using Graphpad Prism v7. Group data are reported as mean ± standard error of the mean (SEM) unless otherwise specified.

Our experimental design followed the following sequence: verification of functional responsiveness of the pelvic nerve to bladder connection; establishing repeated reflex voiding in response to continuous infusion of saline into the bladder; determination of optimal parameters for stimulation of pelvic nerve to inhibit voids whilst producing minimal “off-target” side effects; investigation into the mechanism of action.

## Results

### Effect of low frequency pelvic nerve stimulation

In preliminary experiments (*n* = 3) we tested the effectiveness of low frequency pelvic nerve stimulation in order to assess the functional integrity of the nerve-bladder projection following surgery. In line with data from others (Carpenter and Rubin, 1967; Aronsson et al., 2014) brief trains (10 s) of low frequency stimulation evoked a phasic increase in bladder pressure reflecting contraction of the detrusor (Figure 2A). The effect was dependent on both the intensity (Figure 2B) and the duration (Figure 2C). At low intensities (1–2 V), each pulse evoked a twitch-like response in the EUS EMG (latency = 16–18 ms; Figure 2D). As the stimulation intensity increased, twitch responses were superimposed on tonic EMG activity (Figures 2E,F). Tonic EMG activation was not secondary to increased bladder pressure (Figures 2E,F). Occasionally, a single drop of urine was expelled from the urethra during low frequency stimulation but co-ordinated voids (see below) were never evoked by this procedure.

### Voiding evoked by continuous infusion of saline into the bladder

**Figure 3:**
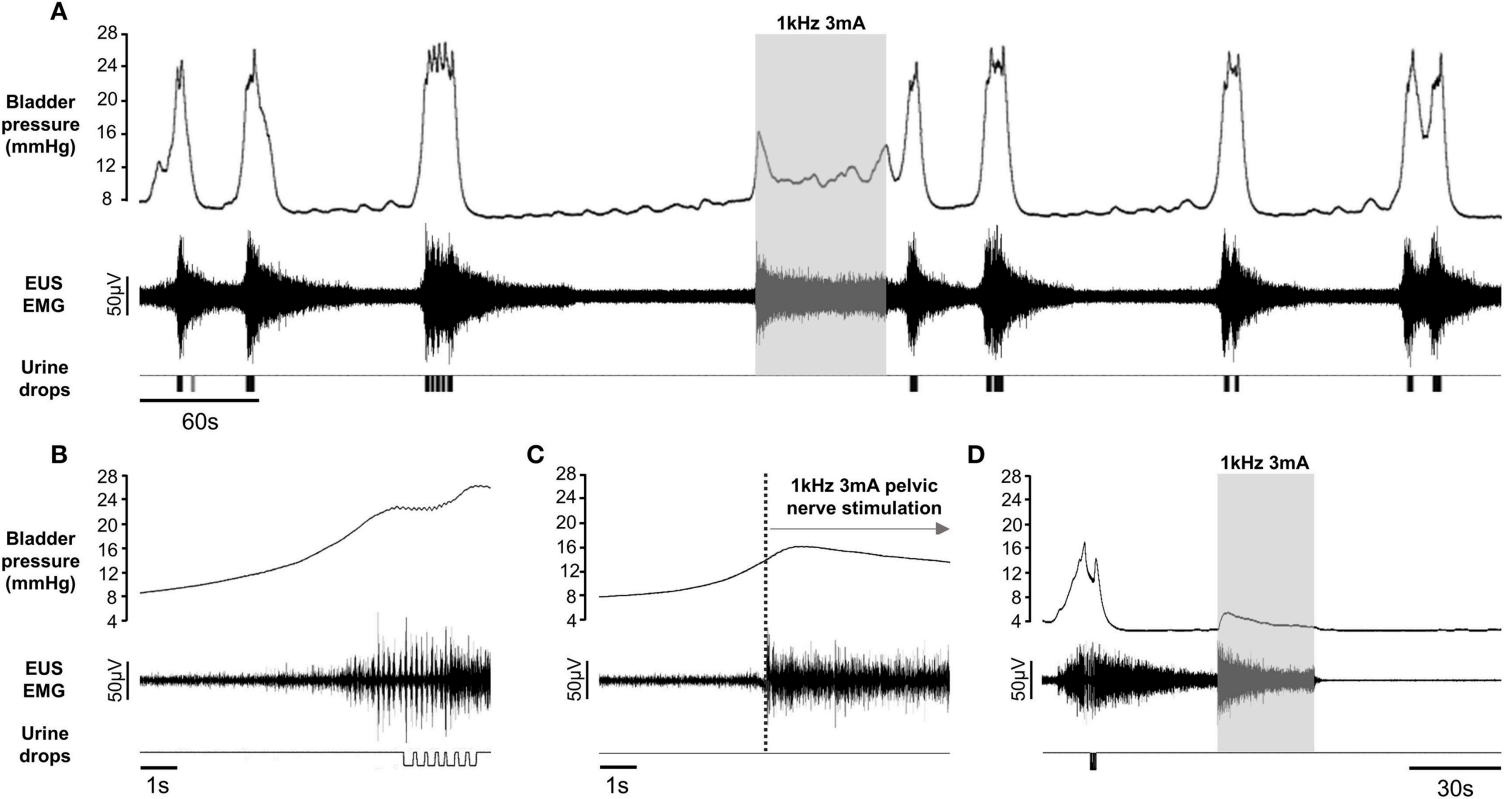
**(A)** Repeated voiding elicited by continuous infusion of saline into the bladder in a urethane anesthetized rat. Initiating pelvic nerve stimulation (gray panel) at the onset of an imminent void indicated by a sharp rise in bladder pressure, aborted the void and urinary continence was maintained during the 1 min period of stimulation, despite continued infusion of saline into the bladder. Voiding resumed once the stimulation was switched off. **(B)** Enlargement of voiding response to illustrate bursting activity in the EUS EMG record during a spontaneous void. **(C)** Sustained tonic activity in EUS during pelvic nerve stimulation. **(D)** Pelvic nerve stimulation during the filling phase evoked sustained tonic EMG activity but only a small transient “on-response” in bladder pressure. All traces from the same rat.

Following the initial exploratory experiments, the correct positioning of the electrode on the pelvic nerve and the functional responsiveness of the detrusor and EUS were confirmed at the start of each new experiment by recording a bladder contraction in response to a short train (10 s) of low frequency square wave stimulation (10 Hz, 1 ms pulses). A stabilization period of 30 min was then allowed before starting continuous infusion of saline into the bladder (6 ml h^−1^). Repeated cycles of filling and voiding were established in 22 out of 30 rats. The onset of each void was characterized by a steep rise in bladder pressure and an increase in tonic EMG activity in the EUS (Figures 3A,B). At the peak of bladder pressure the tonic EMG activity transformed into a bursting pattern (Figure 3B; Stone et al., 2011; Crook and Lovick, 2016) and rhythmic opening and closing of the urethral meatus could be observed as urine was expelled. In contrast to the continuous stream of urine seen in humans in whom the EUS simply relaxes during voiding (Fowler et al., 2008), the rhythmic activity in the EUS of the rat facilitates expulsion of urine via the urethra in spurts. Low amplitude, short-lasting increases in bladder pressure occurred during the filling phase (Figure 2A). These non-voiding contractions were never accompanied by changes in activity in the EUS or by urine output.

### Effect of high frequency pelvic nerve stimulation

**Figure 4:**
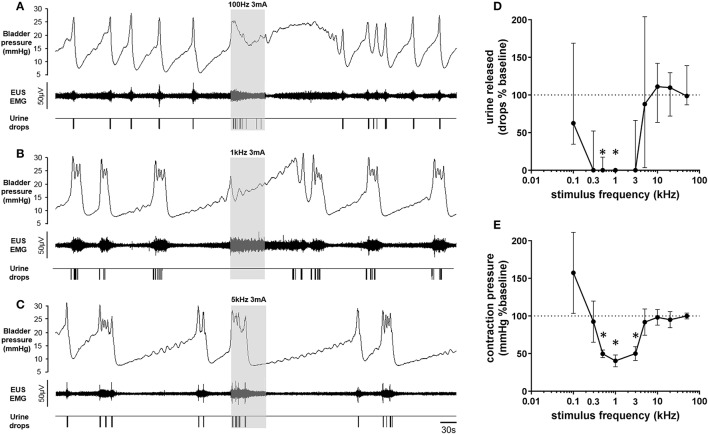
**(A–C)** Frequency dependent effect of 1 min pelvic nerve stimulation on urinary voiding evoked by continuous infusion of saline into the bladder. All data from the same animal. **(D)** Amount of urine released, as a percentage of baseline (mean of 3 previous voids) during the first 30 s of pelvic nerve stimulation at different stimulation frequencies (*n* = 6). Data displayed as median ± interquartile range; ^*^p < 0.05, Wilcoxon signed rank test, *n* = 6. **(E)** Maximum rise in pressure above void threshold during pelvic nerve stimulation expressed as a percentage of baseline void pressure (baseline: mean of 3 previous voids). Data are displayed as mean ± S.E.M., ^*^p < 0.05, Wilcoxon signed rank test, *n* = 6. N.B. Full range of frequencies not tested for each rat, thereby precluding repeated measures comparative testing across all frequencies.

Once established, the pattern of filling and voiding usually continued for several hours, allowing us to investigate the effect of high frequency stimulation of the pelvic nerve (0.5–5 mA; 500 Hz–10 KHz alternating current sinusoidal waveform). When unilateral stimulation of the preganglionic pelvic nerve bundle was initiated within 1 s of the onset of the sharp rise in bladder pressure signaling an imminent void, voiding was inhibited. Bladder pressure ceased rising, tonic activity in the EUS increased and no fluid was expelled from the urethra. We were able to inhibit micturition completely in all but one rat tested (21/22). When stimulation parameters were optimized for each rat, urinary continence was maintained throughout the 1 min period of stimulation despite continuing to infuse saline into the bladder (Figures 3A, 4B). On switching off the stimulation voiding resumed, usually within 1–2 min (Figures 3A, 4A,B). Unilateral stimulation of the left or right side was equally effective, whilst bilateral stimulation (*n* = 3) was no more effective in inhibiting voiding than unilateral stimulation. On occasion, e.g., when stimulation parameters were sub-optimal for the animal, the stimulation suppressed the imminent void, but a void occurred toward the end of the 1 min stimulation period.

High frequency stimulation of the pelvic nerve that suppressed or deferred voiding evoked an increase in tonic activity in the EUS, which was sustained throughout the stimulation period (Figures 3A,C, 4A,B). The bursting activity in the EUS that characterized voiding never developed, regardless of the frequency of stimulation. Stimulating during the filling phase in between voids evoked a small, transient increase in bladder pressure—an “on” response (Figure 3D), together with a sustained increase in the level of tonic activity in the EUS (Figure 3D). No fluid was expelled from the urethra.

To quantify the effect on voiding, the number of drops of urine expelled during the first 30 s of pelvic nerve stimulation initiated at the onset of the rise in pressure signaling an imminent void, was compared to the mean number of drops from the three previous voids (“baseline,” Figure 4D). The effect of pelvic nerve stimulation, tested over the 100 Hz–50 kHz frequency range, keeping intensity for each rat constant (1–5 mA; *n* = 6), revealed a U-shaped response relationship (Figure 4). In every rat stimulation at 1 kHz (1–3 mA intensity) aborted the rise in bladder pressure and no urine was expelled (Figure 4B) whilst stimulation ≥10 kHz was ineffective (Figures 4D,E). Stimulation at interim frequencies (3, 5 kHz) produced variable effects: sometimes the void was deferred until later in the 1 min stimulation period or alternatively, a void occurred but a lower volume of urine was expelled compared to voids that took place in the pre-stimulation baseline period.

**Figure 5:**
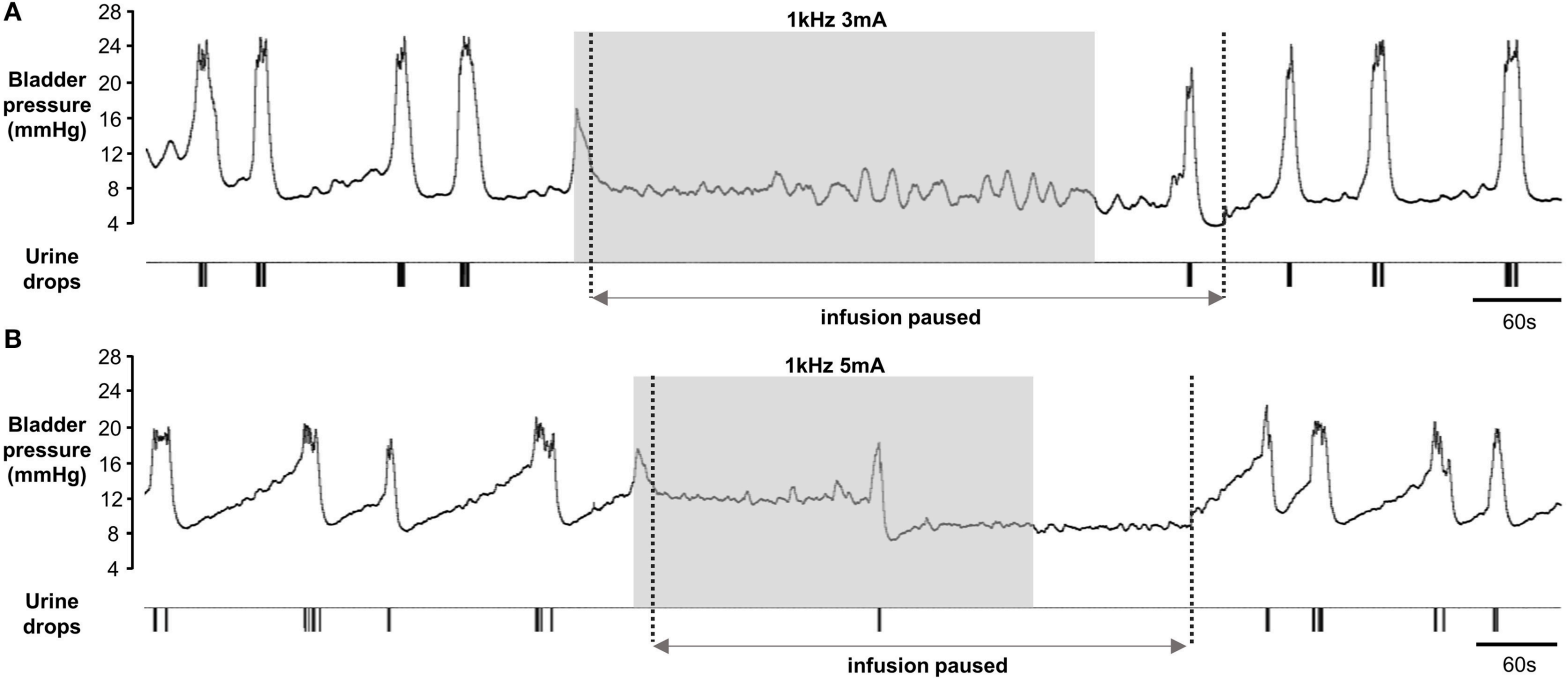
Examples of effect of long periods of pelvic nerve stimulation in 2 rats. Once pelvic nerve stimulation (gray panel) had aborted the void infusion of saline into the bladder was subsequently paused (between broken lines) whilst the stimulation continued. **(A)** Continence was maintained for the full 5 min duration of the stimulation. **(B)** Voiding was deferred for 160 s after onset of pelvic nerve stimulation. Note different intensities of pelvic nerve stimulation were required to inhibit voiding in the different animals.

In four rats we investigated the effects of longer periods of stimulation. We first established parameters for a 60 s period of stimulation to inhibit voiding. Next, we began stimulation at the onset of a rise in bladder pressure indicating an imminent void, waited 10–15 s to check that the void had been aborted, and then halted the infusion of saline into the bladder whilst allowing the stimulator to free run for up to 5 min. In two rats urine output was suppressed completely for the duration of the stimulation (5 min; Figure 5A). In two animals micturition was deferred and a synchronized void occurred 180 and 290 s, respectively, after starting stimulation (Figure 5B). In all rats small phasic increases in bladder pressure were observed during the stimulation period (Figures 5A,B), presumably reflecting non-voiding contractions. There was also sustained tonic activity in the EUS, which was maintained throughout the stimulation (data not shown). When infusion of saline into the bladder recommenced 1–2 min after the end of the stimulation period, periodic voiding was re-established (Figures 5A,B).

### Mechanism of the effect

**Figure 6:**
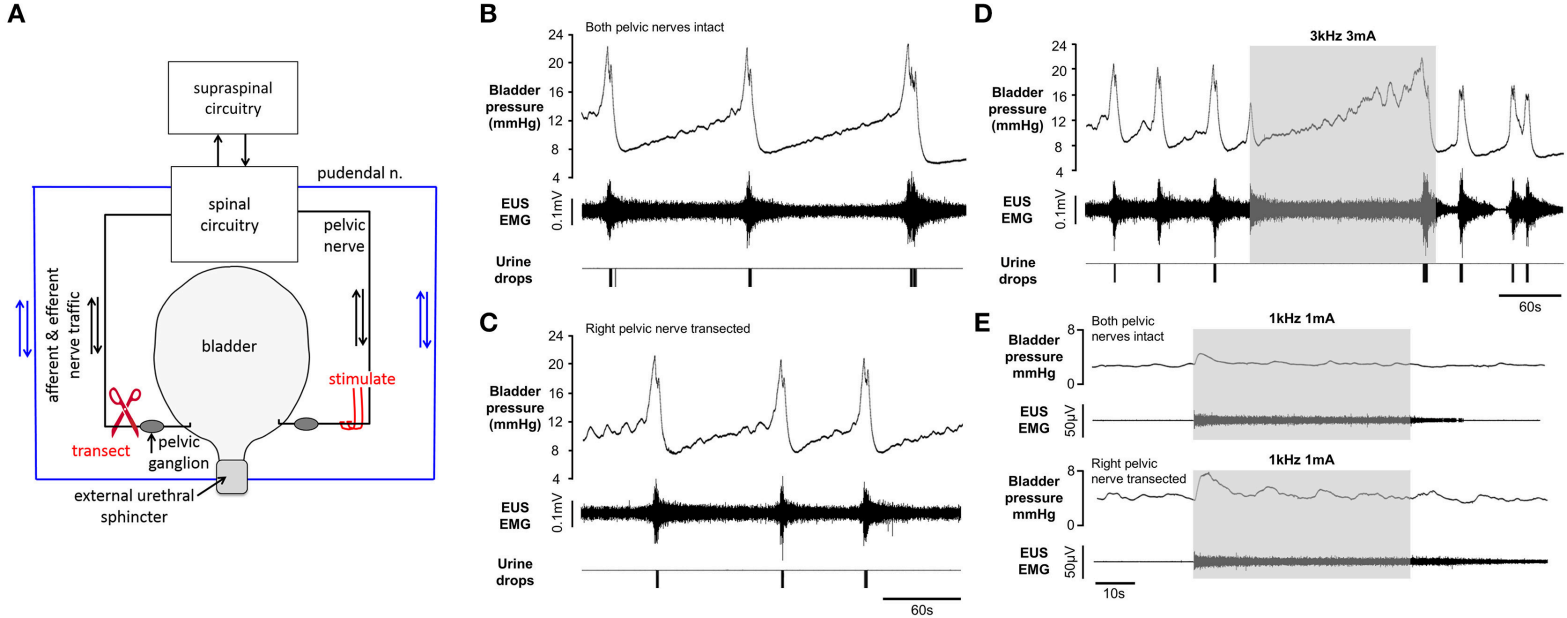
Repeated voiding elicited by continuous infusion of saline into the bladder before **(B)** and after **(C)** unilateral transection of the preganglionic pelvic nerve **(A)** Non-voiding contractions during the filling phase were more prominent following transection of the nerve. **(D)** High frequency pelvic nerve stimulation (gray panel) blocked voiding after contralateral preganglionic pelvic nerve section. **(E)** Pelvic nerve stimulation during the filling phase evoked sustained tonic EMG activity and a transient “on-response” in bladder pressure before (upper traces) and after (lower traces contralateral pelvic nerve section.

Once a stable pattern of voiding had been established in response to continuous infusion of saline into the bladder, we investigated the effect of unilateral preganglionic pelvic nerve transection (*n* = 4; Figure 6A). In three animals the pattern of voiding continued without interruption post-nerve transection (Figures 6B,C). The voiding frequency was unchanged in two of the rats, but in the third animal it increased from 0.42 to 0.93 voids min^−1^. In the remaining rat synchronized voiding ceased following unilateral nerve section; however frequent contractions of the bladder occurred, each accompanied by an increase in tonic EUS activity. Bursting activity, which normally characterizes voiding, did not develop, although a small amount of urine, typically only one drop, was forced out of the urethral meatus during each phasic bladder contraction. In all rats non-voiding contractions present during the filling phase post-unilateral nerve section produced a greater rise in detrusor pressure than before section (1.4 ± 0.4 mm Hg vs. 0.8 ± 0.3 mmHg, respectively; *p* = 0.045 paired *t*-test). Voiding ceased permanently after sectioning both pelvic nerves, although non-voiding contractions, which have been shown by others to be both intrinsically generated and modulated at the level of the pelvic ganglion (Persyn et al., 2016), continued.

In two rats we tested the effect of high frequency pelvic nerve stimulation after sectioning the contralateral nerve. In both rats the inhibitory effect persisted (Figure 6D) and the effect was indistinguishable from the inhibition of voiding evoked in the same animals when both nerves were intact. We also investigated the effect of stimulation during the filling phase before and after contralateral nerve transection. Stimulation under both conditions evoked a small transient rise in bladder pressure (0.5–3.5 mmHg, “on response”) and sustained tonic activation of the EUS (Figure 6E).

**Figure 7:**
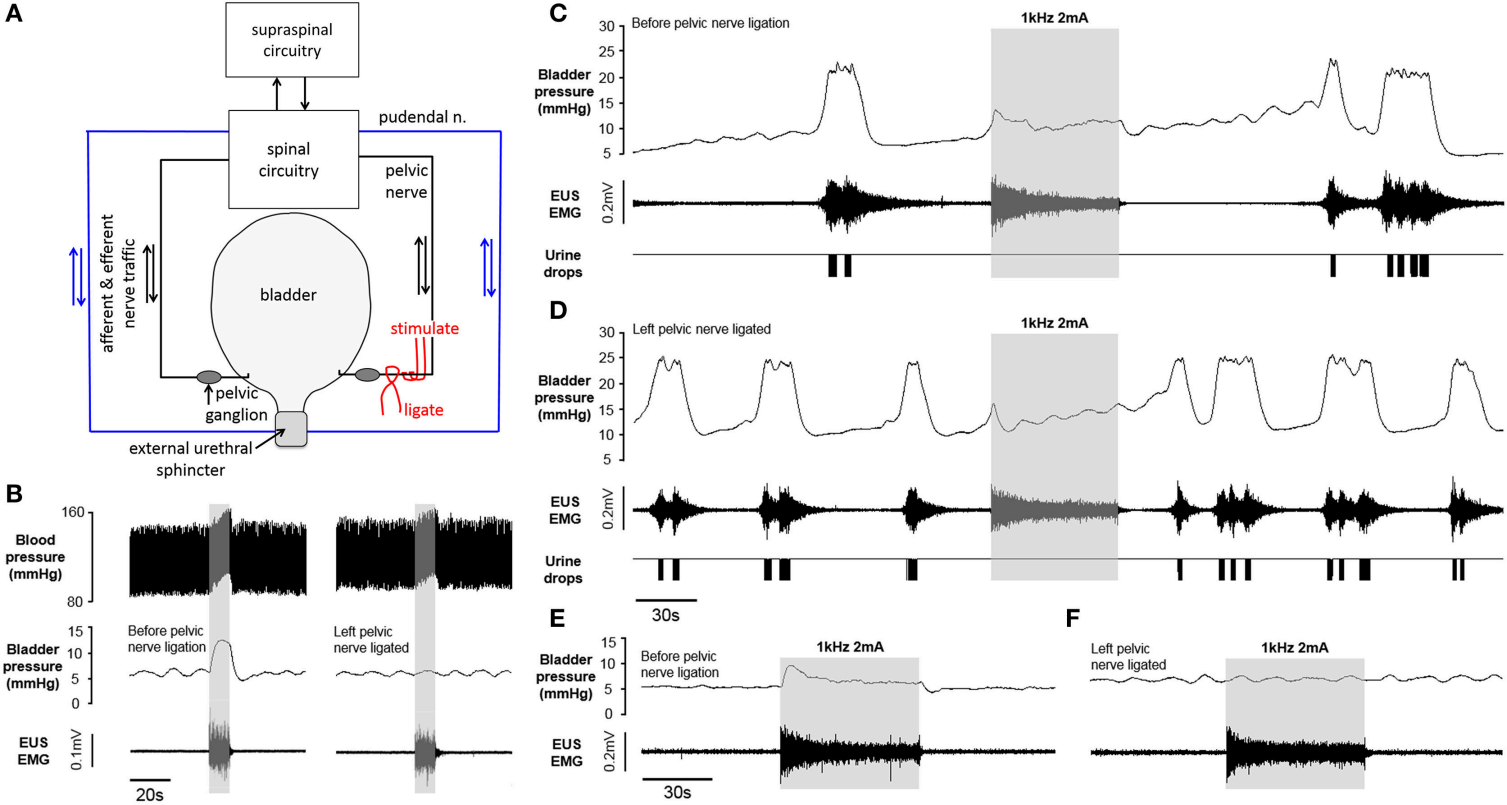
**(A)** Schematic representation of experiments incorporating ipsilateral pelvic nerve ligation. **(B)** Contractile response of the bladder to low frequency stimulation of pelvic nerve (10 Hz, 2 mA, 1 ms pulse width, grey panel) abolished after ipsilateral pelvic nerve ligation. EUS EMG and arterial pressor responses unaffected after nerve ligation. **(C,D)** voiding inhibited during high frequency pelvic nerve stimulation (2 mA 1 kHz sinusoidal, grey panel) after ligation of the distal ipsilateral preganglionic pelvic nerve. **(E,F)** Sustained tonic EMG activity evoked by pelvic nerve stimulation during the filling phase before **(E)** and after **(F)** ipsilateral pelvic nerve ligation. The transient ‘on-response’ in bladder pressure **(E)** was not observed following ligation **(F)**.

In four rats, once we had established that high frequency nerve stimulation inhibited voiding, we investigated the effect of ipsilateral denervation by tightening a loose ligature previously positioned on the nerve between the stimulating electrode and the pelvic ganglion. (Figure 7A). We chose ligation over nerve transection since in initial experiments transecting the nerve lead to the proximal end moving with respect to the stimulating nerve cuff. Complete conduction block post-ligation was verified in three out of four rats by the absence of a rise in bladder pressure in response to a 10 s train of low frequency (10 Hz, 1 ms) square wave pulses (Figure 7B). In the remaining rat the bladder pressure response was reduced to 45% of its magnitude prior to nerve ligation. In all rats the arterial pressor response and increase in EUS activity evoked by the stimulation remained unchanged after nerve ligation (Figure 7B). Following ipisilateral denervation spontaneous voiding in response to infusion of saline into the bladder continued. Void frequency did not change significantly (0.46 ± 0.17 vs. 1.58 0.3 voids min^−1^ pre- and post-ligation, respectively, *p* = 0.08 paired *t*-test). In addition, high frequency stimulation of the proximal end of the pelvic nerve ipsilateral to the ligation (Figure 7A) continued to be effective in blocking voiding (Figures 7C,D). However, the small, transient bladder pressure “on responses” evoked by high frequency pelvic nerve stimulation with the nerve intact, were not observed following ligation (Figures 7E,F).

### “Off target” effects of pelvic nerve stimulation

**Figure 8:**
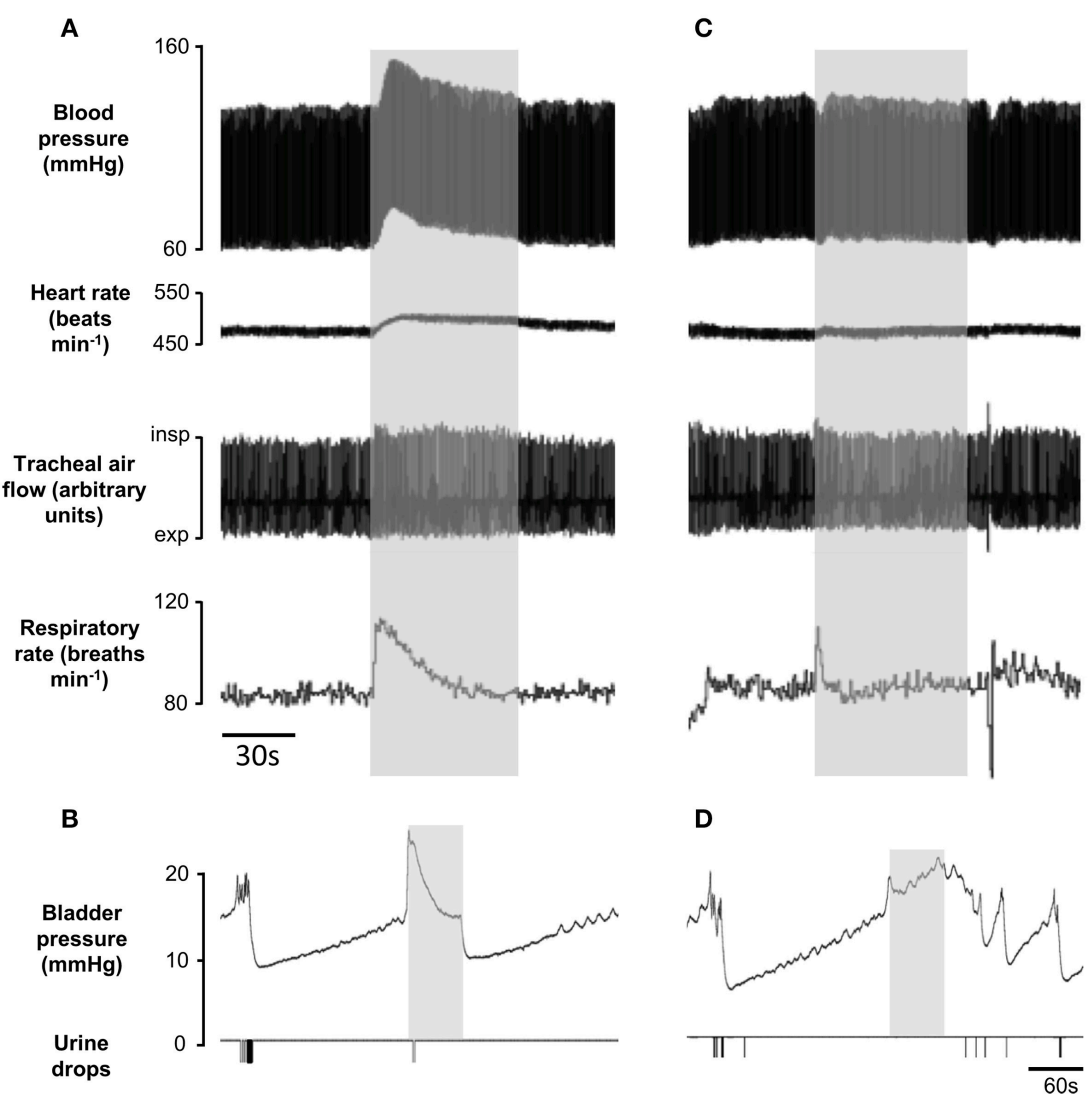
Cardio-respiratory changes evoked by 1 min sinusoidal pelvic nerve stimulation. **(A,B)** stimulation using suboptimal parameters (500 Hz) evoked cardiorespiratory changes and incomplete inhibition of voiding. **(C,D)** stimulation at higher frequency (3 kHz) in the same rat was without effect on blood pressure and heart rate whilst voiding was inhibited completely.

**Figure 9:**
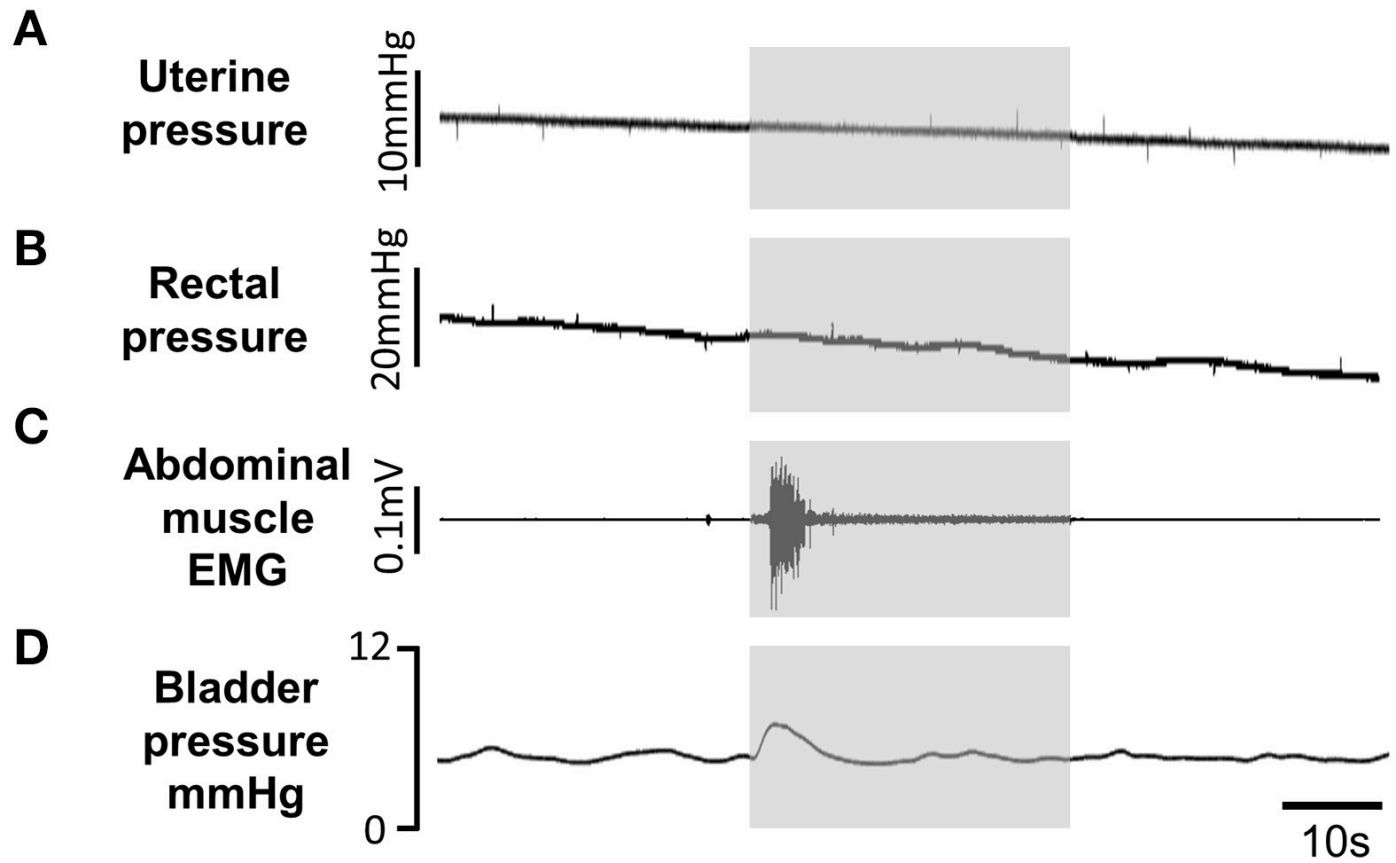
Off-target effects of pelvic nerve stimulation (gray panels) using Stimulation optimal for inhibiting voiding (3 kHz 1 mA sinusoidal waveform) had no effect on uterine **(A)** or rectal **(B)** pressure. A transient contraction of abdominal wall **(C)** was evoked at stimulus onset. Pelvic nerve stimulation between voids evoked only small transient rise in bladder pressure **(D)** at the onset of stimulation. All traces from the same animal.

At the lower end of the effective frequency range for inhibiting or deferring voiding (500 Hz) the stimulus often evoked a sustained rise in bladder pressure as well as a significant rise in blood pressure, tachycardia, and an increase in respiratory rate (Figure 8A). However, in each experiment (*n* = 21), by testing a range of different stimulus intensities and frequencies, we were able to find an optimal parameter for which these cardiorespiratory effects were minimal or prevented completely (Figure 8B), whilst still inhibiting voiding (Figures 8C,D). We were concerned that pelvic nerve stimulation might evoke other “off target” effects. In females, the pelvic nerve runs along the wall of the uterus (Figure 1). This raises the possibility that the stimulus might spread to activate the adjacent uterine muscle. However, no change in uterine pressure was detected during pelvic nerve stimulation that inhibited voiding (*n* = 9; Figure 9A). Neither could we detect any change in rectal pressure (*n* = 5; Figure 9B). Another concern was that pelvic nerve stimulation might induce changes in intra-abdominal pressure. Since a laparotomy had been performed to access the bladder and pelvic nerve, it was not possible to measure intra-abdominal pressure directly. We therefore measured EMG activity in the abdominal wall (*n* = 5) as an index of the abdominal muscle contraction. When using parameters of stimulation that were optimal for blocking urinary voiding there was either no effect (*n* = 2) or just an initial brief “on response” contraction of the abdominal wall at the onset of the stimulation coinciding with the ‘on response’ for bladder pressure (*n* = 3, Figures 9C,D).

## Discussion

In urethane-anesthetized rats continuous infusion of saline into the bladder evoked repeated voiding, in agreement with previous reports (Kruse et al., 1990; Matsuura et al., 2000; Stone et al., 2011; Crook and Lovick, 2016). High frequency stimulation of the pelvic nerve initiated at the onset of an imminent void suppressed voiding. The effect was rapid in onset, reversible and reproducible and when stimulation parameters had been optimized to inhibit voiding, there were only minimal “off target” side effects. This powerful inhibitory effect on voiding was evoked by unilateral pelvic nerve stimulation. Indeed, there was no advantage to be gained by stimulating bilaterally.

In the present study spontaneous voiding in response to infusion of saline into the bladder persisted following unilateral nerve section. Although the bladder is innervated bilaterally, in rats the pelvic nerve trunks on either side each innervate the whole of the bladder (Carpenter and Rubin, 1967). Gap junction coupling between cells (Fry et al., 2004) also enables the detrusor to act as a functional syncytium. Thus, activity in only one pelvic nerve appears to be sufficient to produce a co-ordinated contraction of the detrusor and to initiate reflex bursting activity in the EUS.

Low frequency stimulation of the pelvic nerve evoked a rise in bladder pressure accompanied by contraction of the EUS, in agreement with previous reports (Danziger and Grill, 2016). In contrast, high frequency stimulation of the pelvic nerve, started within 1–2 s of the sharp rise in bladder pressure that signaled an imminent void, aborted the void and no urine escaped. The mechanism underlying this effect is intriguing. In other unmyelinated nerves high frequency stimulation has been shown to produce conduction block (Joseph and Butera, 2009, 2011; Patel and Butera, 2015). However, in the present study nerve block seems unlikely to have been a major underlying factor in inhibiting micturition. Firstly, if a nerve block had occurred under the electrode, the functional integrity of the contralateral nerve should have been sufficient to complete the void. In fact, voiding was suppressed during ipsilateral stimulation even when the contralateral nerve had been sectioned. Secondly, when the ipsilateral nerve was ligated distally, so that the repeated voiding prior to stimulation must have been mediated by activation of the contralateral nerve, high frequency stimulation ipsilaterally was still able to suppress voiding. These factors indicate that the effect of high frequency stimulation was mediated by a signal relayed centrally, which in some way blocked the generation of a motor command signal to the bladder.

Previous studies have shown that low frequency pelvic nerve stimulation (10–20 Hz) can evoke a long-lasting inhibition of spontaneous isovolumetric bladder contractions (De Groat and Ryall, 1968; De Groat, 1976). This effect was due to recurrent inhibition, via crossed and uncrossed pathways, of parasympathetic preganglionic neurons supplying the bladder whose axons were activated antidromically by stimulation of the nerve. However, unlike in the present study, the inhibition could be evoked only when bladder volume and resting pressure were low. This lead the authors to conclude that the effect was unlikely to be operative during the micturition reflex, which is triggered when bladder volume and pressure are relatively high, and more likely functioned during storage as an adjunct to the guarding reflex (De Groat and Ryall, 1968).

High frequency stimulation has been reported to produce axonal conduction block in invertebrate and mammalian nerves (Hulsebosch and Coggeshall, 1982; Bhadra and Kilgore, 2005; Joseph et al., 2007; Joseph and Butera, 2009, 2011; Patel and Butera, 2015). However, effective stimulation frequencies for blocking conduction in unmyelinated fibers (20–30 kHz; Joseph and Butera, 2009, 2011; Patel and Butera, 2015) were much higher than the optimal frequencies (1–3 kHz) that inhibited voiding in our study. Indeed we found stimulus frequencies above 10 kHz to be ineffective. Moreover, since inhibition of voiding persisted after ligation of the pelvic nerve distal to the stimulation site, which blocked conduction to the bladder, it is unlikely that a stimulation-induced blockade of pelvic nerve efferent transmission made a significant contribution to the effects seen in the present study. In support of these findings a recent modeling study (Pelot et al., 2017) predicts that stimulation of small diameter fibers within the range of effective parameters used by us, is likely to excite the nerve and impose on it a firing pattern that is physiologically meaningless with respect to voiding. When transmitted to the spinal cord and supraspinal micturition ccontrol, this would effectively block the circuitry setting up the motor command pattern to generate a void.

High frequency pelvic nerve stimulation that supressed voids, always evoked sustained contraction of the EUS, an effect that undoubtedly contributed toward maintaining continence. The pelvic nerve is a mixed nerve containing small myelinated afferents as well as motor fibers (Hulsebosch and Coggeshall, 1982; Park et al., 1997; Shea et al., 2000; D'Amico et al., 2011). Activation of stretch-sensitive Aδ bladder afferents during bladder filling (Moss et al., 1979) evokes reflex tonic contraction of the EUS: the guarding reflex (Park et al., 1997; D'Amico et al., 2011; Danziger and Grill, 2016). The sustained increase in tonic activity in the EUS during high frequency pelvic nerve stimulation is likely due to activation of these afferent fibers.

Stimulation of pelvic nerve afferents would also be expected to excite the spino-midbrain-spinal micturition control circuit that relays in the midbrain periaqueductal gray and pontine micturition center, which is essential to initiate co-ordinated voiding (De Groat et al., 2015). The mechanosensitive bladder afferents that respond to physiological levels of activation rarely fire in excess of 15 Hz (De Groat and Ryall, 1969; Shea et al., 2000). It is unlikely they would follow faithfully the stimulation in the low kHz frequency range used in the present study. Nevertheless, the stimulation would impose an unphysiological pattern of afferent activity, which would be transmitted to the central micturition control circuitry. This might make it impossible for the circuitry to initiate the co-ordinated activity in the spinal outflows to the detrusor and EUS that are required to produce a void. Whether such an effect would occur within midbrain or spinal levels is not clear. However, electrical stimulation of the midbrain part of this loop has also been shown to be able to block co-ordinated voids (Stone et al., 2015). The authors proposed that by imposing an unphysiological pattern of firing, the stimulus “jammed” the micturition circuitry in the manner that electrical signals are used to jam radio transmission (Stone et al., 2015). A similar mechanism, operating at spinal and/or midbrain level, may have contributed to the functional inhibition of voiding evoked by high frequency pelvic nerve stimulation.

## Conclusion

Notwithstanding uncertainties about the precise underlying mechanism, the present study has demonstrated the ability of high frequency pelvic nerve stimulation to suppress imminent urinary voids. The rapid onset of the effect, its ready reversibility and the absence of significant effects on other organ systems suggests that pelvic nerve stimulation merits consideration as an alternative approach to manage urinary urge incontinence (UUI) in humans. Clinically, UUI may have a diversity of underlying causes. The current experiments were carried out in a rat preparation with no bladder pathology in which we were able to suppress uncontrollable, but essentially normal voiding. It will be important to replicate the findings in other models of UUI which involve bladder pathology.

## Author contributions

TL conceived and designed the experiments with input from JC. JC performed the experiments and analyzed the data with input from TL. TL wrote the first draft of the manuscript, which was subsequently edited by JC and TL. All authors approved the final manuscript.

### Conflict of interest statement

The authors declare that the research was conducted in the absence of any commercial or financial relationships that could be construed as a potential conflict of interest.
